# Sarcoidosis Presenting as Massive Splenic Infarction

**DOI:** 10.1155/2012/834758

**Published:** 2012-07-30

**Authors:** Ishita Patel, Mediola Ismajli, Alan Steuer

**Affiliations:** Rheumatology Department, Wexham Park Hospital, Heatherwood and Wexham Park Hospitals NHS Foundation Trust, Wexham, Slough, Berkshire SL2 4HL, UK

## Abstract

Sarcoidosis is a multisystem granulomatous disease of unknown aetiology. Granulomatous inflammation involving the spleen is common and associated with splenomegaly. However, massive splenomegaly is a rare occurrence. Infrequently massive splenomegaly can result in splenic infarction. Massive splenic infarction in sarcoidosis has, to our knowledge, not been previously reported. We present a case of a woman presenting with massive splenic infarction and sarcoidosis confirmed by granulomatous inflammation of the liver.

## 1. Introduction

Sarcoidosis is an idiopathic chronic systemic granulomatous disease. It commonly presents with pulmonary granulomatous disease but extrapulmonary manifestations also occur with varying frequency. Granulomatous infiltration of the spleen is common in sarcoidosis but is often asymptomatic. Splenomegaly is unusual, and massive splenomegaly leading to splenic infarction is very rare.

## 2. Case Presentation

A 53-year-old lady was admitted under the surgical team with a six-month history of progressive left-sided abdominal pain, associated with anorexia and lethargy. She reported no other systemic symptoms of note. Her past medical history consisted of mild depression and recurrent sinusitis over a 20-year period. Clinical examination revealed skin pallor and a palpable left upper quadrant mass.

**Figure 1:**
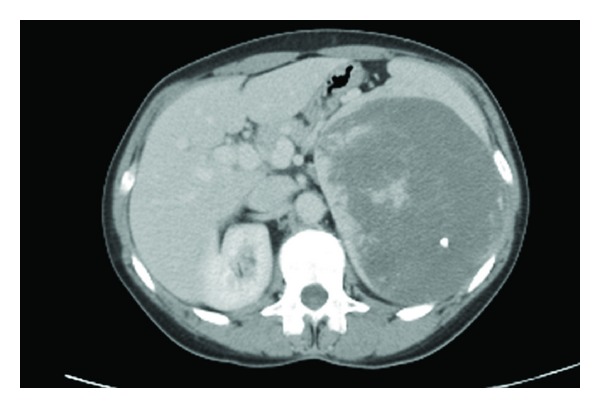
CT image of large left upper quadrant mass with evidence of necrosis and a calcific focus.

Investigations including a full blood count and biochemical profile were normal. Her chest radiograph was normal. An abdominal ultrasound revealed a well-defined heterogeneous solid mass with flecks of calcification in the left upper quadrant. Abdominal CT scan revealed a large well-defined, solid thick-walled necrotic mass with calcific foci within the left upper quadrant. The mass measured approximately 13 cm by 11 cm in size (Figure 1). There was no lymphadenopathy, nor focal liver abnormality.

The patient underwent a laparotomy, which revealed the mass was a grossly enlarged spleen. A splenectomy was performed. The liver appeared macroscopically abnormal. A liver biopsy was undertaken. The patient had an unremarkable postoperative recovery.

Histological examination of the spleen revealed massive global parenchymal infarction with some periarteriolar fibrosis. The liver biopsy, showed multiple noncaseating granulomas. Further histological assessment excluded any evidence of fungal or mycobacterial infection.

Given the appearance of florid granulomatous change in the liver, the patient was referred to our rheumatology department. Further investigations showed a normal serum ACE, negative TB ELISPOT, and negative ANA, ENA, and ANCA. A normal ferritin and serum immunoglobulin levels and negative serology for hepatitis A, B, and C were noted. Serology for brucellosis, histoplasmosis, and leishmaniasis were negative. A gallium scan revealed intense uptake solely within the liver.

A diagnosis of sarcoidosis of the liver with associated massive splenic infarction was made. The patient was not commenced on any treatment. She is under regular review and remains asymptomatic two years after presentation. Follow up ultrasound scans of her liver and liver function tests remain normal.

## 3. Discussion

Sarcoidosis is a multisystem granulomatous disease of unknown aetiology, most commonly affecting the lungs in 90% of cases and lymph nodes (particularly intrathoracic), followed by liver 50–80%, skin 25%, and eyes 11–83%; other organs are less frequently involved [1]. Granulomatous infiltration of the spleen is common in sarcoidosis, but splenic enlargement is unusual and massive splenomegaly is rare. In a large review by Fordice et al. of 6074 cases of sarcoidosis, 628 patients had quantifiable splenomegaly and only 3% had massive splenomegaly [2]. There have been a few cases reported in the literature of massive splenomegaly in sarcoidosis [3–5]. Thirty to 60% of cases of splenic involvement in sarcoidosis are asymptomatic.

Computed tomography of the abdomen is very useful in evaluating splenic sarcoidosis, which typically manifests as homogeneous organomegaly. However, there are a few reported cases of nodular splenic sarcoidosis and still fewer having massive splenomegaly with low-attenuation nodules [6]. Scintigraphy with gallium-67 scanning provides a better way of assessing granulomatous lesions in sarcoidosis not revealed by traditional methods of investigation. A study by Beaumont et al. evaluated the usefulness of gallium-67 scanning in 54 patients with sarcoidosis. They found that gallium-67 scan was effective in detecting and assessing lesions particularly those affecting the mediastinum, spleen, and salivary glands [7].

Splenic infarction occurs as a result of vascular compromise to the organ. Common causes include thromboembolism and infiltrative haematological diseases that cause congestion of the splenic circulation with abnormal cells. The mechanism of massive splenic infarction in our patient is unknown. Patients with sarcoidosis have been shown to have impaired vascular endothelial function and increased arterial stiffness according to a study by Siasos et al. of eighty-seven patients with sarcoidosis [8]. Conceivably, chronic perivascular inflammation resulting in the periarteriolar fibrosis seen on histology may have compromised vascular supply, leading to visceral ischaemia and infarction.

Our case demonstrates a rare presentation of massive splenic infarction in a patient with liver sarcoidosis. This contributes to the heterogeneity of clinical manifestations of this disease of unknown aetiology.
